# Impact of perioperative α1-antagonists on postoperative urinary retention in orthopaedic surgery: meta-analysis

**DOI:** 10.1093/bjsopen/zrac144

**Published:** 2023-01-06

**Authors:** Yun-Ting Huang, Yu Chang, Yi-No Kang, Chin-Hsuan Huang, Yu-Shiuan Lin, Jeffrey Wu, Kuan-Yu Chi, Wei-Cheng Chen

**Affiliations:** Department of Anesthesiology, National Cheng Kung University Hospital, College of Medicine, National Cheng Kung University, Tainan, Taiwan; Department of Surgery, Section of Neurosurgery Department, National Cheng Kung University Hospital, National Cheng Kung University, Tainan, Taiwan; Department of Education, Center for Evidence-Based Medicine, Taipei Medical University Hospital, Taipei, Taiwan; Department of Education, Center for Evidence-Based Medicine, Taipei Medical University Hospital, Taipei, Taiwan; Department of Emergency Medicine, Taipei Tzu Chi Hospital, Buddhist Tzu Chi Medical Foundation, New Taipei City, Taiwan; Department of Orthopaedics, Shuang Ho Hospital, Taipei Medical University, Taipei, Taiwan; Department of Education, Center for Evidence-Based Medicine, Taipei Medical University Hospital, Taipei, Taiwan; Department of Internal Medicine, Taipei Medical University Hospital, Taipei, Taiwan; Department of Orthopaedics, Shuang Ho Hospital, Taipei Medical University, Taipei, Taiwan

## Abstract

**Background:**

Postoperative urinary retention (POUR) is a common complication following orthopaedic surgery. Previous studies attempted to establish the preventative role of α1-antagonist in POUR in the general surgical population; however, there is still no consensus regarding its use in orthopaedic surgery due to limited evidence.

**Methods:**

Electronic databases of Cochrane Library, Embase, MEDLINE, and ClinicalTrials.gov were searched by two independent investigators from inception to 1 March 2022 to identify relevant randomized clinical trials. Two reviewers independently completed a critical appraisal of included trials by using the Cochrane Risk of Bias tool version 2.0 and extracted data from included articles. Risk of POUR was summarized as risk ratio (RR) with 95 per cent confidence intervals (c.i.). Mean difference (MD) was used for meta-analysis of continuous outcomes.

**Results:**

Five randomized clinical trials involving 878 patients (α1-antagonist, 434; placebo, 444) undergoing hip/knee arthroplasty and spine surgeries were included. One study was assessed as high risk of bias from the randomization process and was excluded from the final meta-analysis. There was no difference in the risk of POUR between patients taking α1-antagonist and the placebo in arthroplasty (RR, 0.64; 95 per cent c.i., 0.36 to 1.14) and in spine surgeries (RR, 1.03; 95 per cent c.i., 0.69 to 1.55). There was no difference in length of stay (MD, −0.14 days; 95 per cent c.i., −0.33 to 0.05). Use of α1-antagonist was associated with a higher risk of adverse events (RR, 1.97; 95 per cent c.i., 1.27 to 3.06), with a composite of dizziness, light-headedness, fatigue, altered mental status, and syncope being the most commonly reported symptoms.

**Conclusion:**

In patients undergoing spinal surgery and joint arthroplasty, routine administration of perioperative α1-antagonist does not decrease risk of POUR but does increase perioperative dizziness, light-headedness, and syncope.

## Teaser

In patients undergoing spine surgeries and joint arthroplasty, routine administration of perioperative α1-antagonist does not confer to a decrease in the risk of postoperative urinary retention but a significant increase in the risk of perioperative dizziness, light-headedness, and syncope.

## Introduction

Postoperative urinary retention (POUR) is the inability to void and is a common complication resulting from orthopaedic surgery. Based on a recent meta-analysis of 29 352 elective spine operations performed between 2001 and 2020, the incidence of POUR was 15.1 per cent.^1^ A systematic review of 6397 patients, enrolled from 2011 to 2019, for joint arthroplasty reported incidence of POUR that varied from 5.5 per cent to 46.3 per cent^2^. The incidence of POUR in both types of surgery is significantly higher than the estimated incidence of 3.8 per cent in the general surgical population^3^. Although intermittent urinary catheterization is the standard management for POUR, it can cause patient discomfort, urethral trauma, and catheter-related urinary tract infection (CRUTI)^4^. For orthopaedic operations, CRUTI is a predisposing factor for prosthetic joint infection and osteomyelitis, which lengthen the hospital stay and can potentially compromise the surgical outcomes due to bacteraemia^5,6^. There are several unmodifiable risk factors, including old age, male sex, benign prostate hyperplasia, and diabetes mellitus^1,2^, highlighting the need for interventions to reduce POUR.

Many trials have investigated the role of α1-antagonist in the prevention of POUR based on the presumptive overactivation of α1-adrenergic receptor across the bladder neck and urethra^3–7^; however, the results from different types of surgery have been contradictory^8–12^. Although two recent meta-analysis have demonstrated the preventive role of α1-antagonist in patients undergoing primary unilateral inguinal hernia repair^13^, and several types of operations^14^, the results were presented with a considerable amount of heterogeneity. Moreover, only one meta-analysis^14^ included an orthopaedic cohort, which has led to an uncertainty regarding the use of α1-antagonist in orthopaedic surgery. Our aim was therefore to investigate the preventative effects of α1-antagonist on the orthopaedic surgical population by performing a systematic review with meta-analysis.

## Methods

We conducted the systematic review and meta-analysis based on the Cochrane Handbook for Systematic Reviews of Interventions^15^ and reported results based on the Preferred Reporting Items for Systematic Reviews and Meta-Analyses (PRISMA) statement^16^ (*Appendix S1*). The study was registered on PROSPERO (CRD42022311809).

### Study selection

Two investigators (Y.T.H. and Y.C.) independently searched electronic databases of PubMed, Embase, Cochrane Library and ClinicalTrials.gov from inception up until 1 March 2022, to identify relevant studies. Any discrepancy was addressed by reaching a consensus with senior reviewers (W.C.C.). Search details are presented in *Appendix S2*.

### Eligibility criteria

Four predefined criteria for evidence selection were as follows: randomized clinical trials (RCTs) to avoid confounding bias from observational studies; studies involving adult patients aged more than 18 years who underwent any orthopaedic surgery with the prescription of perioperative α1-antagonist; studies with clear definition of POUR; and studies reporting the comparative postoperative outcomes, at least including POUR, between α1-antagonist and placebo.

### Data extraction

Two investigators (Y.T.H. and Y.S.L.) independently extracted relevant information from eligible articles, including first author’s name; publication year; country; study interval; sample size; surgery type; protocol of perioperative α1-antagonist administration, including dose and days; definition of POUR; demographics of participants: patients’ age, sex, co-morbidities (for example hypertension, diabetes mellitus (DM), and benign prostatic hyperplasia (BPH)), and opioid use; and adverse events posed by α1-antagonist and placebo. Any discrepancy was addressed by reaching a consensus with senior reviewers (K.Y.C.).

### Quality assessment

Two investigators (Y.C. and K.Y.C.) independently completed a critical appraisal of included trials by using the Cochrane Risk of Bias tool version 2.0 (ROB 2.0)^17^. Any discrepancy was addressed through discussion with senior author W.C.C.

### Main outcomes and statistical analysis

Meta-analysis was conducted using RStudio (*Appendix S3*) with the ‘metafor’ package^18^. Through the inverse variance method and the Mantel-Haenszel method, we pooled continuous and binary outcomes and the resuts were summarized using mean difference (MD) and risk ratios (RRs) respectively. The primary outcome was RR of POUR between the intervention group and the placebo group (risk of POUR in the intervention group *versus* risk of POUR in the placebo group). Secondary outcomes included the difference in length of stay (LOS) and risk of adverse events between the use of α1-antagonist and the placebo. Those available data from included studies were pooled and analysed through random-effects meta-analysis with the restricted maximum likelihood method^19^ being used as a heterogeneity estimator because between-trial variance was inevitable. Heterogeneity was assessed using *I*^20^, with values of *I*^2^ less than 25 per cent, more than 25 per cent and lower than 50 per cent, and more than 50 per cent indicating low, moderate, and high heterogeneity respectively. Determination of statistical significance in these analyses followed a common threshold (*P* < 0.050). All the outcome parameters are presented with a 95 per cent c.i. and *P* value. In studies with high heterogeneity, sensitivity analyses through either subgroup analysis or meta-regression, to further investigate possible clinical and methodological sources of heterogeneity was used. If pooling was unavailable, the qualitative synthesis method was undertaken. For potential publication bias, funnel plot and Egger’s test was used if the study number was less than 10.

## Results

**Fig. 1:**
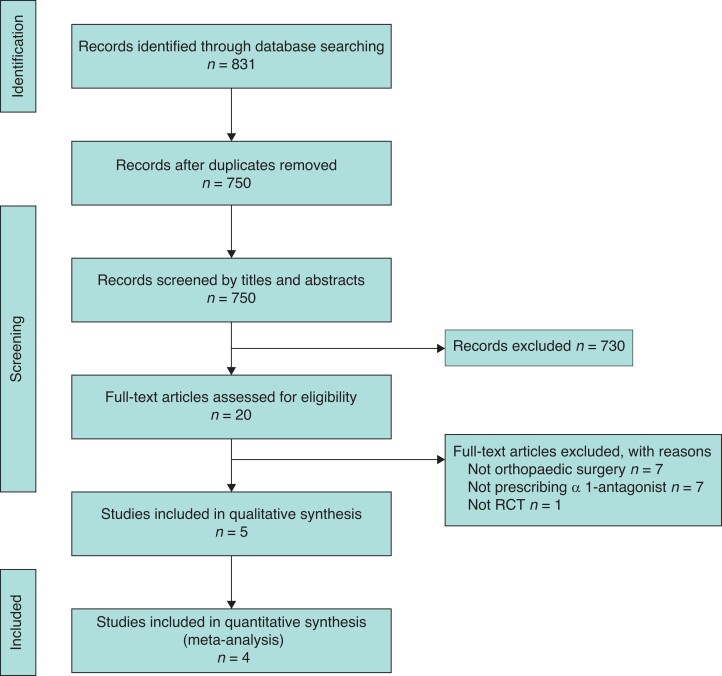
PRISMA flowchart diagram RCT, randomized clinical trial.

The study identified 831 references, with 20 studies for full-text review. Fifteen studies did not meet eligibility criteria. Five studies^10,11,0–2^ were identified for qualitative and quantitative syntheses (*Fig. 1*).

### Characteristics of included studies

Five RCTs^10,11,1–3^ included 878 patients (α1-antagonist, 434; placebo, 444) who were enrolled between 1987 and 2019 (*Table 1*). Of the five RCTs, three^11,21,22^ involved patients receiving primary arthroplasty and two^10,20^ involved patients undergoing elective spine surgery. In the study by Petersen *et al*.^23^, prazosin was prescribed as perioperative α1-antagonist in the intervention group, whereas the other four studies used tamsulosin.

**Table 1:** Study characteristics

Study	Rughani et al.^10^	Basheer et al.^21^	Choi et al.^22^	Schubert et al.^11^	Petersen et al.^23^
**Country**	USA	USA	South Korea	USA	USA
**Study interval**	2016–2019	2012–2013	2018	2017	1987–1989
**Surgery type**	Elective spine surgery	Elective spine surgery	Primary total hip or knee arthroplasty	Primary total hip or knee arthroplasty	Primary total hip or knee arthroplasty
**α1-antagonist prescription**	Preoperative: 5-day	Preoperative: 2-day	Preoperative: 3-day	Preoperative: 5-day	Preoperative: 1 mg prazosin 48 h before surgery, then 2 mg every 12 h if tolerated Postoperative: 2 mg every 12 h during hospitalization
	tamsulosin 0.4 mg QD	tamsulosin 0.4 mg QD	tamsulosin 0.2 mg QN	tamsulosin 0.4 mg QD	
	Postoperative: 2-day tamsulosin 0.4 mg QD	Postoperative: 1-day tamsulosin 0.4 mg QN	Postoperative: 3-day tamsulosin 0.2 mg QN	Postoperative: 1-day tamsulosin 0.4 mg QD	
**POUR definition**	Inability to void 6 h after surgery or development of bladder discomfort, with PVR > 300 ml	PVR > 250 ml after the voiding trial	PVR > 400 ml after the voiding trial	(1) PVR volume > 200 ml after indwelling urinary catheter removal; (2) Bladder discomfort or distention causing inability to void regardless of PVR; (3) Requirement of intermittent straight catheterization during hospitalization	Inability to void when bladder became distended
**Study arm**	α1-antagonist Placebo	α1-antagonist Placebo	α1-antagonist Placebo	α1-antagonist Placebo	α1-antagonist Placebo
**Study size** **(Male per cent)**	245 (100) 252(100)	49 (100) 46 (100)	48 (31.3) 47 (29.8)	64 (100) 67 (100)	28 (21.4) 32 (59.4)
**Mean age (s.d)**	63.4 (7.7) 63.7 (8.7)	57.7 (15.1) 57.0 (13.9)	65.8 (16.8) 70.8 (14.4)	60.55 (10.4) 61.37 (9.6)	63.8 (NA) 66.7 (NA)
**LOS (days)**	0.7 0.9	3.5 2.8	NA NA	1.13 1.21	NA NA

LOS, length of stay; NA, not applicable; POUR, postoperative urinary retention; PVR, post-void residual volume; QD, once daily; QN, once every night.

### Risk of bias assessments

**Fig. 2:**
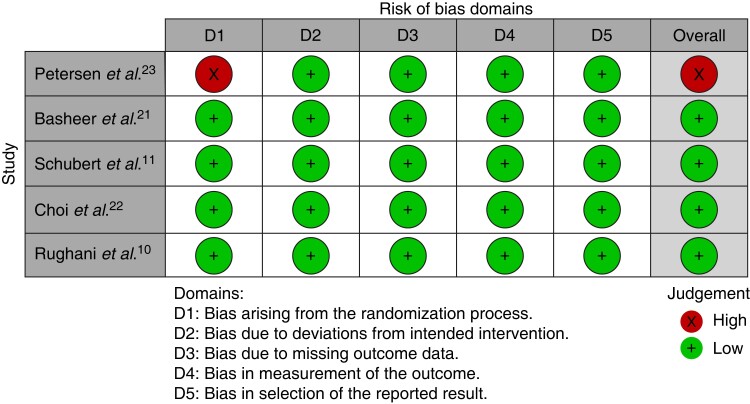
Visual summary of risk of bias using ROB 2.0 evaluation tool Four included trials are evaluated as low overall risk of bias and one as high overall risk of bias.

Quality assessments of the included trials with ROB 2.0 are summarized in *Fig. 2*. Four included trials^10,11,20,21^ were evaluated as having a low risk of bias throughout all domains. There was a high risk of bias in one study due to a bias in randomization.

### Primary outcome: risk ratio of POUR in α1-antagonist *versus* placebo

**Fig. 3:**
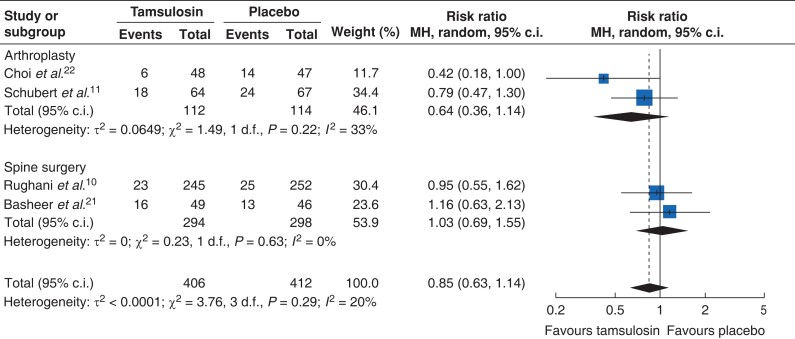
Forest plot of POUR Forest plot demonstrates the risk ratio of POUR between α1-antagonist and the placebo. POUR, postoperative urinary retention.

We included four studies^10,11,21,22^ with low risk of bias (*Fig. 3*). All the included studies, except for Basheer *et al*.^21^, performed power analysis and achieved the targeted sample size. Overall, the pooled result indicated the use of perioperative α1-antagonist conferred a comparable risk of POUR to placebo, with low statistical heterogeneity (RR, 0.85; 95 per cent c.i., 0.63 to 1.14, *I*^2^ = 20 per cent; *Fig. 3*). Notably, after the subgroup analysis based on surgical types, the risk of POUR between the two arms remained similar in both patients receiving arthroplasty (RR, 0.64; 95 per cent c.i., 0.36 to 1.14; *I*^2^ = 33 per cent; *Fig. 3*) and spine surgery (RR, 1.03; 95 per cent c.i., 0.69 to 1.55; *I*^2^ = 0 per cent; *Fig. 3*).

### Secondary outcomes: LOS and adverse events between α1-antagonist and the placebo

**Fig. 4:**
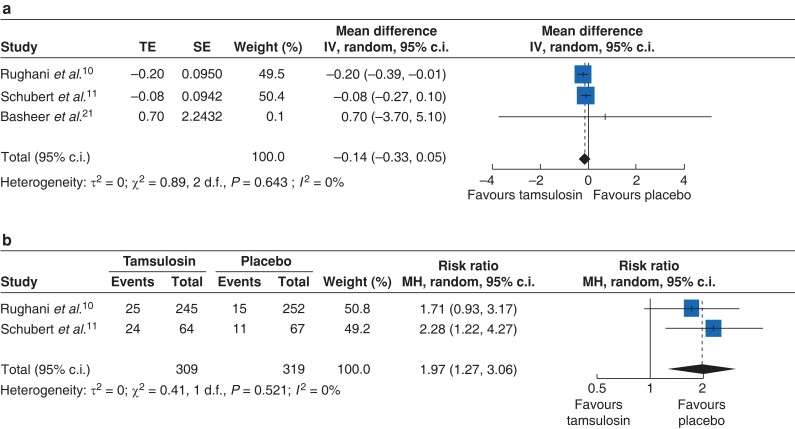
Forest plot of LOS and adverse effect **a** Forest plot demonstrates the difference in LOS between α1-antagonist and the placebo. **b** Forest plot demonstrates the risk ratio of α1-antagonist-related adverse events between tamsulosin and the placebo. LOS, length of stay.

The pooled results of three studies showed no significant difference in LOS between the intervention and control group (MD, −0.14; 95 per cent c.i., −0.33 to 0.05; *I*^2^ = 0 per cent; *Fig. 4a*); however, the use of α1-antagonist caused a significantly higher risk of overall adverse events than the placebo (RR, 1.97; 95 per cent c.i., 1.27 to 3.06; *I*^2^ = 0 per cent; *Fig. 4b*). *Table 2* lists the details of side effects reported in two studies. The main contributing side effects of α1-antagonist are dizziness, light-headedness, fatigue, syncope, and altered mental status. Notably, floppy iris syndrome, a unique event exclusive to tamsulosin, was found in two cases that involved the use of the α1-antagonist.

**Table 2:** Adverse events

Adverse event	Tamsulosin (*n* = 309)	Placebo (*n* = 319)
Floppy iris syndrome	2 (0.6)	0 (0)
Dizziness/light-headedness/syncope	13 (4.2)	3 (0.9)
Hypotension	0 (0)	1 (0.3)
Constipation	2 (0.6)	1 (0.3)
Nausea/emesis	5 (1.6)	1 (0.3)
Headache	2 (0.6)	0 (0)
Others*	27 (8.7)	19 (6.0)

Values are *n* (%). *Other adverse events included: infection, hives/pruritus, gout flare, delayed wound healing, calf pain, and gastrointestinal bleeding.

## Discussion

In contrast with the beneficial effect of α1-antagonist on herniorrhaphy^13^, colporrhaphy^24^, and urological surgeries^8,9,25^, our study showed that the α1-antagonist does not confer a decrease in the risk of POUR but actually confers an increase in the overall drug-related adverse events after spinal surgery and primary knee/hip arthroplasty. There is evidence suggesting that POUR is primarily driven by α-adrenergic overstimulation of bladder neck muscles during and following surgery, dysregulation of β2 receptor, γ-aminobutyric acid receptor, and prostaglandin inhibition also play a pivotal role^4,26^. Orthopaedic surgery differs in a number of ways from general surgery, including patient positioning, perioperative use of narcotics, and surgical instruments^27^. α-1 overstimulation might not be the main cause of POUR in orthopaedic surgery. For instance, a prone position, one of the most frequently used approaches in spine surgery, commonly results in high intra-abdominal pressure due to the thoracoabdominal compression, which can further increase intraoperative bleeding, induce haemodynamic instability, lengthen the operating time, and reduce cardiac output during the operation^1,28–30^. A prone position can increase the requirement for intraoperative fluid, causing bladder distension and possible POUR^31^. Joint arthroplasty has several factors, such as age, male sex, and spinal anaesthesia that are recognized as significant contributors to POUR^2^.

Our meta-analysis of RCTs contradicts the results of a propensity-matched retrospective study that demonstrated a significant 12.1 per cent decrease in the risk of POUR^32^. Although the study controlled various confounding factors through a propensity score and an adjusted analysis, a myriad of risk factors may be undetected due to the nature of the retrospective study, further contributing to unmeasured confounding bias^33^. For instance, co-morbidities such as diabetes, previous cerebral vascular accidents, and long-term medication use of anti-cholinergics and beta-blockers can lead to a predisposition to POUR^4^ and were unmeasured and remained uncontrolled in the study. Records of intermittent catheterization after removal of an intraoperative urinary catheter during postoperative hospitalization was used as a definition but lacked standardization. It is difficult to validate the window of perioperative administration of the α1-antagonist simply through medical records, and any documented use of the medication has the potential to be outside of the perioperative interval, which may give rise to selection bias.

POUR has a significant influence on the LOS and Rughani *et al*.^10^ demonstrated an mean stay of 0.66 days in patients without POUR compared with 1.73 days in those with POUR^10^; however, there are several factors that contribute to increased LOS, including Foley catheterization, complexity of the operation, and co-morbidities, and our study indicates that using α1-antagonist does not significantly decrease LOS when compared with a placebo. These findings indirectly suggest that the α1-antagonist has a neutral effect on orthopaedic cohorts in terms of both POUR and LOS. With respect to drug-related side effects, although headache, nausea, vomiting, constipation, and other adverse events listed in *Table 2* could be confounded by hospital admission and perioperative interval, the fact that the use of α1-antagonist was associated with a significant risk of dizziness, light-headedness, and syncope are not unexpected and can be attributed to the physiological action of α-1 blockage. Notably, Schubert *et al*.^11^ reported two cases of floppy iris syndrome, a known side effect exclusive to tamsulosin, which causes pupil dilation and quivering of the iris^34^.

Although our results are presented with low statistical heterogeneity, one may argue that there is a potential source of conceptual heterogeneity that may be undetected numerically and that is due to the diverse definition of POUR.

Throughout the literature review, many criteria have been proposed for the diagnosis of POUR (for example patient discomfort from palpable distended bladder^35,36^, micturition score^37^, inability to void with bladder distention^22^, or inability to void 8 h after Foley removal^38^), bladder catheterization, and ultrasound assessment using post-void residual (PVR) volume cut-off, with no single one considered to be the standard. Most studies included in our analysis, except for Petersen *et al.*, utilized PVR volume after the voiding trial. The normal capacity of a bladder in the adult population ranges from 400 to 600 ml^2^. Reaching a volume of 150 ml in the bladder creates the first urge to void and, notably, a sense of fullness is perceived as the tension receptors are activated when the volume reaches approximately 300 ml^4^. Any volume above 600 ml is considered to be pathological. The cut-off used in included studies ranges from 200 to 400 ml after the voiding trial, and one prospective study^39^ demonstrated that the threshold of 200 ml was useful in predicting the development of POUR (sensitivity, 0.80 and specificity, 79 per cent). Therefore, we do not assume the cut-off to be a significant source of heterogeneity as the range matches the physiological threshold of a sense of fullness and should be considered as a normal variation.

Two previous meta-analyses have established the protective role of α1-antagonist in POUR, with Clancy *et al*.^13^ investigating the hernia repair population, and Ghuman *et al*.^14^ investigating the general surgical population, irrespective of surgery type. Despite the latter meta-analysis exploring a broad spectrum of operations, only one included study^23^ enrolled an orthopaedic cohort, which represented a small fraction of the entire study. The results were presented with significant heterogeneity including multiple types of α1-antagonists, including prazosin and phenoxybenzamine, with each possessing different selectivity of α-1 receptors compared with tamsulosin. Clancy *et al*.^13^ included five RCTs with three (Gönüllü *et al*.^40^, Woo *et al*.^12^, and Goldman *et al*.^41^) investigating prazosin and phenoxybenzamine, and it was suggested that, based on visualization of the forest plot, the data from Woo *et al*.^12^ and from Goldman *et al*.^41^ were the outliers of the pooled results. This phenomenon, to some extent, reflects the aforementioned difference in activity and specificity between prazosin/phenoxybenzamine and tamsulosin.

This study has several limitations. First, although our findings seem reassuring and informative for spine surgery and primary knee/hip arthroplasty, we were unable to generalize our results to other orthopaedic surgery, such as arthroscopy, bone cancer, and trauma surgery. Second, the protocol for the perioperative use of α1-antagonists varies between studies. Other commonly used α1-antagonists, such as alfuzosin and doxazosin, are unavailable in current RCTs. The types of perioperative anaesthesia and the use of postoperative analgesia have a pivotal role in the development of POUR; however, the paucity of detailed information across included studies limits the assessment. Although BPH is a strong predictor for male patients developing POUR, the diagnosis of BPH in included studies was established based on medical records or chart reviews and there was a lack of assessment on the severity of the BPH (for example the International Prostate Symptom Score or uroflowmetry). All studies in our meta-analysis, except for Choi *et al*.^22^ only included male patients, which may also compromise the generalizability to the orthopaedic population.

## Supplementary Material

zrac144_Supplementary_Data

## Data Availability

The authors confirm that the data supporting the findings of this study are available within the article and its Supplementary material.
