# Supplementary material for: Impact of perioperative α1-antagonists on postoperative urinary retention in orthopaedic surgery: meta-analysis
Source: BJS Open. 2023 Jan 6;7(1):zrac144. doi: 10.1093/bjsopen/zrac144 (PMC9825733; doi:10.1093/bjsopen/zrac144)
Supplement: zrac144_Supplementary_Data [file zrac144_supplementary_data.docx]

**Title**

**Impact of perioperative α1-antagonists on postoperative urinary retention in orthopaedic surgery: meta-analysis**

Yun-Ting Huang^1^, M.D.†, Yu-Chang^2^, M.D.†, Yi-No Kang^3^, M.A.,
Yu-Shiuan Lin^4^, M.D., Jeffrey Wu, M.D^5^., Kuan-Yu Chi^3,6^, M.D.*, Wei-Cheng, Chen^5^ M.D.*

^1^Department of Anesthesiology, National Cheng Kung University Hospital, College of Medicine, National Cheng Kung University, Tainan, Taiwan.

^2^Department of Surgery, Section of neurosurgery department, National Cheng Kung University Hospital, National Cheng Kung University, Tainan, Taiwan

^3^Department of Education, Center for Evidence-Based Medicine, Taipei Medical University Hospital, Taipei, Taiwan
^4^Department of Emergency Medicine, Taipei Tzu Chi Hospital, Buddhist Tzu Chi Medical Foundation, New Taipei City, Taiwan

^5^Department of Orthopedics, Shuang Ho Hospital, Taipei Medical University, Taipei, Taiwan

^6^Department of Internal Medicine, Taipei Medical University Hospital, Taipei, Taiwan

†Authors equally contributed to the manuscript

***Corresponding author**

Kuan-Yu Chi, M.D.

Department of Internal Medicine, Taipei Medical University Hospital, Taipei, Taiwan

Department of Education, Center for Evidence-Based Medicine, Taipei Medical University Hospital, Taipei, Taiwan

Email: [kuanyuhippo@gmail.com](mailto:kuanyuhippo@gmail.com)

Wei-Cheng Chen, M.D.

Department of Orthopedics, Shuang Ho Hospital, Taipei Medical University, Taipei, Taiwan

Email: [p830113@gmail.com](mailto:p830113@gmail.com)

**Supplementary Materials - Index**

**Appendix S1.** Preferred Reporting Items for Systematic Reviews and Meta-Analyses (PRISMA) 2020 checklist *pag. 4-8*

**Appendix S2.** Search strategy *pag. 9-11*

**Appendix S3.** Data synthesis *pag. 12-13*

**Appendix S1.** PRISMA 2020 checklist

| **Section and Topic** | **Item #** | **Checklist item** | **Location where item is reported** |
| --- | --- | --- | --- |
| **TITLE** | | |  |
| Title | 1 | Identify the report as a systematic review. | Page 1 |
| **ABSTRACT** | | |  |
| Abstract | 2 | See the PRISMA 2020 for Abstracts checklist. | Page 3, 4 |
| **INTRODUCTION** | | |  |
| Rationale | 3 | Describe the rationale for the review in the context of existing knowledge. | Page 5, 6 |
| Objectives | 4 | Provide an explicit statement of the objective(s) or question(s) the review addresses. | Page 5, 6 |
| **METHODS** | | |  |
| Eligibility criteria | 5 | Specify the inclusion and exclusion criteria for the review and how studies were grouped for the syntheses. | Page 7 |
| Information sources | 6 | Specify all databases, registers, websites, organisations, reference lists and other sources searched or consulted to identify studies. Specify the date when each source was last searched or consulted. | Page 6 |
| Search strategy | 7 | Present the full search strategies for all databases, registers and websites, including any filters and limits used. | eMethod 3 |
| Selection process | 8 | Specify the methods used to decide whether a study met the inclusion criteria of the review, including how many reviewers screened each record and each report retrieved, whether they worked independently, and if applicable, details of automation tools used in the process. | Page 6 |
| Data collection process | 9 | Specify the methods used to collect data from reports, including how many reviewers collected data from each report, whether they worked independently, any processes for obtaining or confirming data from study investigators, and if applicable, details of automation tools used in the process. | Page 7 |
| Data items | 10a | List and define all outcomes for which data were sought. Specify whether all results that were compatible with each outcome domain in each study were sought (e.g. for all measures, time points, analyses), and if not, the methods used to decide which results to collect. | Page 8 |
|  | 10b | List and define all other variables for which data were sought (e.g. participant and intervention characteristics, funding sources). Describe any assumptions made about any missing or unclear information. | Page 8 |
| Study risk of bias assessment | 11 | Specify the methods used to assess risk of bias in the included studies, including details of the tool(s) used, how many reviewers assessed each study and whether they worked independently, and if applicable, details of automation tools used in the process. | Page 8 |
| Effect measures | 12 | Specify for each outcome the effect measure(s) (e.g. risk ratio, mean difference) used in the synthesis or presentation of results. | Page 8 |
| Synthesis methods | 13a | Describe the processes used to decide which studies were eligible for each synthesis (e.g. tabulating the study intervention characteristics and comparing against the planned groups for each synthesis (item #5)). | Page 6 |
|  | 13b | Describe any methods required to prepare the data for presentation or synthesis, such as handling of missing summary statistics, or data conversions. | Page 8 |
|  | 13c | Describe any methods used to tabulate or visually display results of individual studies and syntheses. | eMethod 4 |
|  | 13d | Describe any methods used to synthesize results and provide a rationale for the choice(s). If meta-analysis was performed, describe the model(s), method(s) to identify the presence and extent of statistical heterogeneity, and software package(s) used. | eMethod 4 |
|  | 13e | Describe any methods used to explore possible causes of heterogeneity among study results (e.g. subgroup analysis, meta-regression). | Page 8 |
|  | 13f | Describe any sensitivity analyses conducted to assess robustness of the synthesized results. | Page 8 |
| Reporting bias assessment | 14 | Describe any methods used to assess risk of bias due to missing results in a synthesis (arising from reporting biases). | Page 9 |
| Certainty assessment | 15 | Describe any methods used to assess certainty (or confidence) in the body of evidence for an outcome. | N/A |
| **RESULTS** | | |  |
| Study selection | 16a | Describe the results of the search and selection process, from the number of records identified in the search to the number of studies included in the review, ideally using a flow diagram. | Page 9 |
|  | 16b | Cite studies that might appear to meet the inclusion criteria, but which were excluded, and explain why they were excluded. | Figure 1 |
| Study characteristics | 17 | Cite each included study and present its characteristics. | Page 9 |
| Risk of bias in studies | 18 | Present assessments of risk of bias for each included study. | Page 9 |
| Results of individual studies | 19 | For all outcomes, present, for each study: (a) summary statistics for each group (where appropriate) and (b) an effect estimate and its precision (e.g. confidence/credible interval), ideally using structured tables or plots. | Figure 3 |
| Results of syntheses | 20a | For each synthesis, briefly summarise the characteristics and risk of bias among contributing studies. | Figure 2 |
|  | 20b | Present results of all statistical syntheses conducted. If meta-analysis was done, present for each the summary estimate and its precision (e.g. confidence/credible interval) and measures of statistical heterogeneity. If comparing groups, describe the direction of the effect. | Page 10 |
|  | 20c | Present results of all investigations of possible causes of heterogeneity among study results. | Page 10 |
|  | 20d | Present results of all sensitivity analyses conducted to assess the robustness of the synthesized results. | Page 9 |
| Reporting biases | 21 | Present assessments of risk of bias due to missing results (arising from reporting biases) for each synthesis assessed. | N/A |
| Certainty of evidence | 22 | Present assessments of certainty (or confidence) in the body of evidence for each outcome assessed. | N/A |
| **DISCUSSION** | | |  |
| Discussion | 23a | Provide a general interpretation of the results in the context of other evidence. | Page 11 |
|  | 23b | Discuss any limitations of the evidence included in the review. | Page 16 |
|  | 23c | Discuss any limitations of the review processes used. | Page 16 |
|  | 23d | Discuss implications of the results for practice, policy, and future research. | Page 16 |
| **OTHER INFORMATION** | | |  |
| Registration and protocol | 24a | Provide registration information for the review, including register name and registration number, or state that the review was not registered. | Page 6 |
|  | 24b | Indicate where the review protocol can be accessed, or state that a protocol was not prepared. | Page 6 |
|  | 24c | Describe and explain any amendments to information provided at registration or in the protocol. | Page 6 |
| Support | 25 | Describe sources of financial or non-financial support for the review, and the role of the funders or sponsors in the review. | N/A |
| Competing interests | 26 | Declare any competing interests of review authors. | N/A |
| Availability of data, code and other materials | 27 | Report which of the following are publicly available and where they can be found: template data collection forms; data extracted from included studies; data used for all analyses; analytic code; any other materials used in the review. | N/A |

**Appendix S2.** Search strategy

**Embase**

| Search Number | Search Description | Numbers of results |
| --- | --- | --- |
| 1 | (Alfuzosin OR doxazosin OR terazosin OR tamsulosin OR silodosin OR naftopidil or Phenoxybenzamine):ti,ab,kw,de | 30415 |
| 2 | (Alpha block* or alpha antag*):ti,ab,kw,de | 62296 |
| 3 | 'alpha adrenergic receptor blocking agent'/exp | 328852 |
| 4 | Postop* | 1328654 |
| 5 | ((Urin* or void* or bladder) and (reten* or retain* or difficulty or dysfunction)):ti,ab,kw,de | 129785 |
| 6 | 'urine retention'/exp | 31833 |
| 7 | (1 OR 2 OR 3) AND 4 AND (5 OR 6) and [embase]/lim | 555 |

**Medline**

| Search Number | Search Description | Numbers of results |
| --- | --- | --- |
| 1 | (Alfuzosin OR doxazosin OR terazosin OR tamsulosin OR silodosin OR naftopidil or Phenoxybenzamine).mp | 20551 |
| 2 | (Alpha block* or alpha antag*).mp | 11703 |
| 3 | Exp "Adrenergic alpha-Antagonists"/ | 53252 |
| 4 | (Postop*).mp | 932522 |
| 5 | ((Urin* or void* or bladder) and (reten* or retain* or difficulty or dysfunction)).mp | 60044 |
| 6 | exp "urinary retention"/ | 4938 |
| 7 | (1 OR 2 OR 3) AND 4 AND (5 OR 6) | 129 |
| [mp=title, abstract, original title, name of substance word, subject heading word, floating sub-heading word, keyword heading word, organism supplementary concept word, protocol supplementary concept word, rare disease supplementary concept word, unique identifier, synonyms] | | |

**Cochrane library**

**
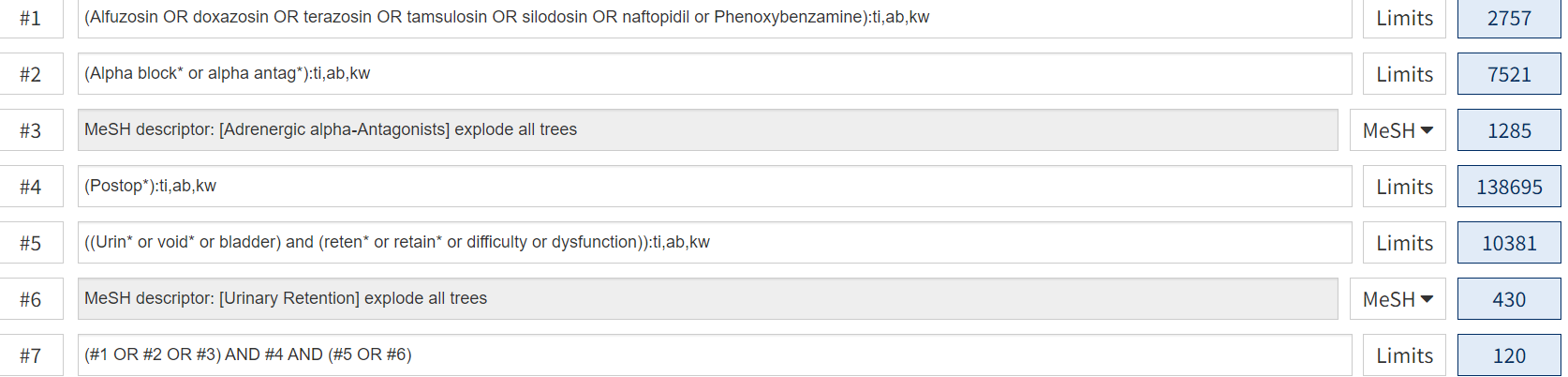
**

**ClinicalTrials.gov**


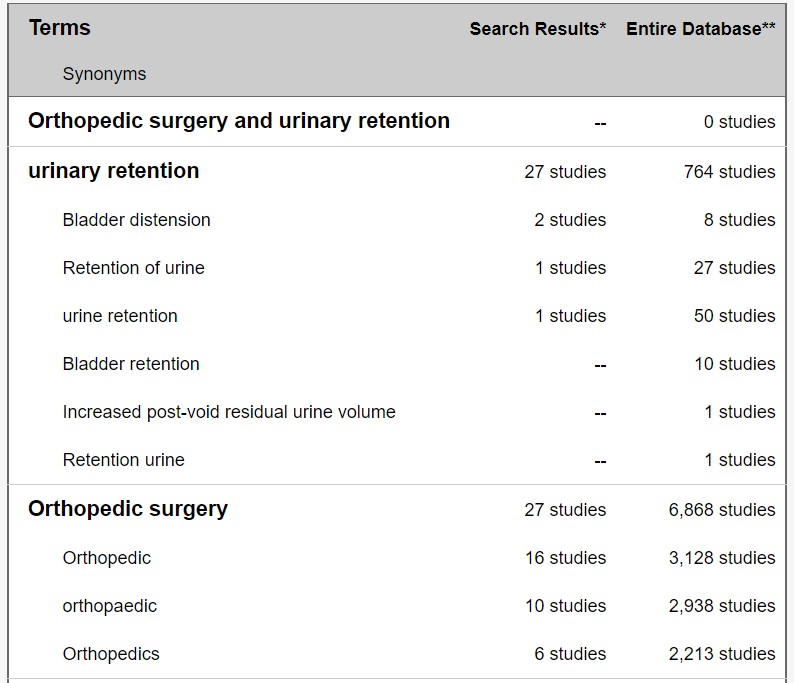


**Appendix S3.** Data synthesis

We used Rstudio with metafor packages to conduct statistical analysis**【metafor】package**
**Random-effects model**
**Generic inverse variance meta-analysis (metagen)**

**Restricted maximum likelihood (REML) method as a heterogeneity estimator**

**Several plots for meta-analysis: Forest plot (forest)**

**Primary outcome**

***Risk of POUR***

metaresult=metagen(logRR, selogRR, studlab=year,sm="RR",data=POUR, method.tau="REML",comb.random=TRUE,comb.fixed=FALSE)

***Subgroup analysis***

metaresult=metagen(logRR, selogRR, studlab=year,sm="RR",data=POUR, method.tau="REML",comb.random=TRUE,comb.fixed=FALSE, byvar= surgery)

***Forest plot***

forest (metaresult, layout="RevMan5", lab.e="α1-antagonist ", lab.c="Placebo", xlab="Favors α1-antagonist Favors placebo ", ff.xlab="bold", col.by="black", pooled.events=F, comb.random=T, comb.fixed=F, col.diamond.random=("red"), col.diamond.lines.random="red", col.square = 'blue', col.square.lines = 'blue')

**Secondary outcome**

***LOS***

metaresult<-metacont(n.e, mean.e, sd.e,n.c, mean.c, sd.c,studlab=year,sm="WMD",data=LOS,method.tau="REML",comb.random=TRUE,comb.fixed=FALSE)

***Risk of Adverse event***

metaresult=metagen(logRR, selogRR, studlab=year,sm="RR",data=Adverseevent, method.tau="REML",comb.random=TRUE,comb.fixed=FALSE)

***Forest plot***

forest (metaresult, layout="RevMan5", lab.e="α1-antagonist ", lab.c="Placebo", xlab="Favors α1-antagonist Favors placebo ", ff.xlab="bold", col.by="black", pooled.events=F, comb.random=T, comb.fixed=F, col.diamond.random=("red"), col.diamond.lines.random="red", col.square = 'blue', col.square.lines = 'blue')
